# 3D Bioprinted Nanocellulose-Based Hydrogels for Tissue Engineering Applications: A Brief Review

**DOI:** 10.3390/polym11050898

**Published:** 2019-05-17

**Authors:** Sandya S. Athukoralalage, Rajkamal Balu, Naba K. Dutta, Namita Roy Choudhury

**Affiliations:** Chemical and Environmental Engineering, School of Engineering, RMIT University, Melbourne, Victoria 3000, Australia; sandya.athukoralalage@rmit.edu.au (S.S.A.); rajkamal.balu@rmit.edu.au (R.B.)

**Keywords:** nanocellulose, 3D printing, hydrogels, biocompatibility, tissue engineering

## Abstract

Nanocellulosic materials, such as cellulose nanocrystals, cellulose nanofibers, and bacterial nanocellulose, that display high surface area, mechanical strength, biodegradability, and tunable surface chemistry have attracted great attention over the last decade for biomedical applications. Simultaneously, 3D printing is revolutionizing the field of biomedical engineering, which enables the fast and on-demand printing of customizable scaffolds, tissues, and organs. Nanocellulosic materials hold tremendous potential for 3D bioprinting due to their printability, their shear thinning behavior, their ability to live cell support and owing to their excellent biocompatibility. The amalgamation of nanocellulose-based feedstocks and 3D bioprinting is therefore of critical interest for the development of advanced functional 3D hydrogels. In this context, this review briefly discusses the most recent key developments and challenges in 3D bioprinting nanocellulose-based hydrogel constructs that have been successfully tested for mammalian cell viability and used in tissue engineering applications.

## 1. Introduction

Hydrogels are three-dimensional (3D) networks of crosslinked hydrophilic polymer chains, which are capable of imbibing large quantities of water [1]. Among polymers of natural origin, cellulose is the most abundant, renewable, inexpensive, and readily available polysaccharide in the world with an annual production of 10^11^ to 10^12^ tons [2]. The major sources of cellulose are plant fibers and wood. Cellulose is a linear polymer, consisting of D-anhydroglucose units joined together by β-1,4-glycosidic linkage, that can exist as microfibrils of different crystalline polymorphs (I, II, III and IV) [3]. Cellulose I is the natural or native form of cellulose, which is the crystal structure that has the highest axial elastic modulus, is thermodynamically metastable, and can be converted to either cellulose II or III. Cellulose I is found in two distinct crystal phases: Iα (triclinic), predominantly found in algae and bacteria, and Iβ (monoclinic), predominantly found in bacteria, plants, and tunicates. Cellulose II is the most stable crystal structure of technical relevance and can be produced by the regeneration and mercerization of cellulose I [3].

**Figure 1:**
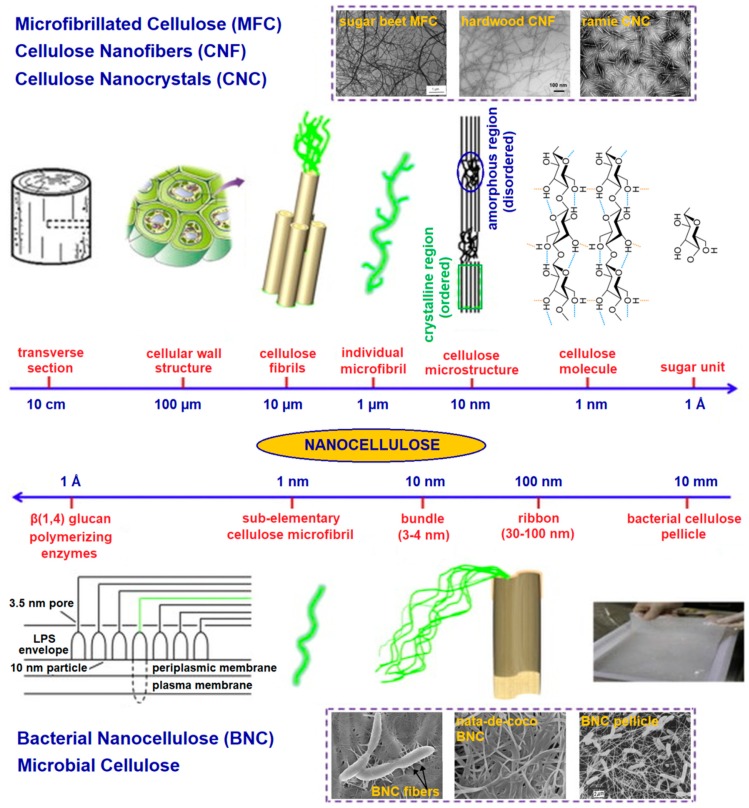
Hierarchical structure of cellulose: top image: plant cellulose; bottom image: bacterial cellulose. Adapted with permission from ref. [5]. Copyright 2014, Elsevier.

During biosynthesis, intermolecular hydrogen bonds between hydroxyl and oxygen groups of adjacent molecules; and van der Waals forces promote parallel stacking of multiple cellulose chains, forming stable elementary fibrils with high axial stiffness that further aggregate into larger microfibrils (5–50 nm in diameter and several microns in length), as shown in Figure 1 [3,4,5]. These cellulose fibrils consist of highly ordered (crystalline) and disordered (amorphous-like) regions that are the main reinforcement segment for plants, trees, algae, and some marine creatures and bacteria [3]. Although bacterial cellulose has the same molecular formula as plant cellulose, its macromolecular properties differ from plant cellulose and exhibit characteristic ribbon-like microfibrils [6]. Cellulose-based hydrogels are of wide scientific interest for their applications in multidisciplinary areas, such as agriculture, textiles, energy, biomedical, etc. [7]. 

Over the last decade, hydrogels made from nanocellulosic materials, such as cellulose nanowhiskers or nanocrystals (CNCs), cellulose nanofibrils or nanofibers (CNFs), and bacterial nanocellulose (BNC) have attracted great attention for biomedical applications owing to their high surface area, high mechanical strength, tunable surface chemistry, excellent biocompatibility, cellular recognition, and biodegradability [8]. 

## 2. Nanocellulose: Synthesis, Mechanical Properties, Biodegradation and Biocompatibility

CNCs are highly crystalline needlelike cellulose nanostructures of 10–20 nm in width and several hundred nanometers in length, whereas CNFs and BNC are long, flexible cellulose fibers with a high aspect ratio, and consist of both crystalline and amorphous regions with a fibril diameter similar to, or larger than, CNCs [3]. CNCs and CNFs are largely sourced from plants and trees, whereas BNC is mainly generated by cultured gram-negative bacteria *Gluconacetobacter xylinus* [5]. CNCs are commonly obtained by strong acid hydrolysis of native cellulose fibers, using sulfuric or hydrochloric acid [4]. CNFs are isolated through high-pressure, high temperature, high velocity impact homogenization, and grinding or the microfluidization process [9]. Lately, (2,2,6,6-Tetramethylpiperidin-1-yl)oxyl (TEMPO)-mediated oxidation coupled with low speed mechanical treatment is becoming increasingly popular for CNF isolation. This method uses the TEMPO radical as a catalyst to selectively oxidize the primary alcohol groups in the cellulose leaving CNFs with a carboxylic acid surface [10]. Conversely, BNC can be produced in a static culture, which results in the accumulation of a thick, white nanocellulose pellicle (Figure 1) at the air–liquid interface and/or stirred culture, which results in dispersed nanocellulose (as irregular pellets or suspended fibers) in the culture medium [6]. 

Nanocrystalline cellulose with a density of 1.6 g/cm^3^ has demonstrated a high tensile strength of 7.5–7.7 GPa (twice that of Kevlar fiber), elastic modulus of 110–220 GPa (in the range of steel wire) in the axial direction, and elastic modulus of 10–50 GPa (greater than carbon nanotubes) in the transverse direction [3]. Moreover, the reactive surface (–OH side groups) of nanocellulose facilitates grafting chemical species to achieve different surface properties that facilitate self-assembly, controlled dispersion within a wide range of polymer matrix, controlled particle-matrix and particle-particle bond strength, and directed cellular response [3]. Nanocellulose has been successfully employed as reinforcing agents in many biodegradable polymers and hydrogels owing to their high mechanical strength and tunable surface chemistry properties [11,12,13]. 

The biodegradation of nanocellulose is commonly performed by cellulolytic microorganisms, which produce enzymes (endoglucanases, cellobiohydrolases, and β-glucosidases) that act together in synergy and catalyze the depolymerization of cellulose [14]. In the typical enzymatic hydrolysis of nanocellulose, endoglucanases and cellobiohydrolases are responsible for degrading cellulose to cellobiose, which is further hydrolyzed to free glucose molecules by β-glucosidases [14,15]. Moreover, γ-irradiation has been reported to induce scission of the long glucan chains, which results in reduced crystallinity and molecular weight, and enhances the degradation rate of nanocellulose [16]. Recently, Singh et al. [17] studied the biodegradation of nanocrystalline cellulose by two environmentally-relevant consortia, and compared it with microcrystalline cellulose. The authors reported that the sulfuric acid hydrolyzed nanocellulose degraded faster than microcrystalline cellulose, and that nanocellulose was more biodegradable, but likely via different pathways. 

Evaluation of nanocellulose in both in vitro and in vivo conditions with different cell lines have demonstrated non-immunogenicity and no to low cytotoxicity (in some cases at low concentration) responses. However, CNFs’ surfaces that were modified with polyethyleneimine and cetyl trimethylammonium bromide showed somewhat cytotoxic effects to mouse fibroblast cells. BNC is commonly believed to possess better biocompatibility than other types of nanocellulose, where no foreign body reaction observed when introduced in animals is well demonstrated [5]. Moreover, the cell behaviors have not only been demonstrated to be affected by the hydrogel’s physical and chemical properties, but also by its 3D geometrical structures that mimic the native extracellular matrix (ECM) environment. To date, several approaches including homogenization, cyclic freeze-thaw, free radical polymerization, UV/ion mediated cross-linking and 3D printing have been reported for the preparation of nanocellulose-based hydrogels for biomedical applications [18]. 

## 3. 3D Bioprinting Approach for Hydrogel Fabrication

3D printing, also known as additive manufacturing or rapid prototyping, is a process for constructing 3D physical objects from digital models through the successive layer-by-layer deposition of materials such as metals, ceramics, polymers and/or living cells [19]. The two most common technologies used in 3D printing are stereolithography (SLA), where the solid part is produced from liquid or ink by polymerization; and fused deposition modeling (FDM), where a continuous filament of thermoplastic is used to form hardened continuous layers [20]. 

The applications of 3D printing in the field of biomedical engineering can be divided into four main areas: (i) manufacturing of permanent non-bioactive implants, (ii) fabrication of local bioactive and biodegradable scaffolds, (iii) manufacturing pathological organ models to aid preoperative planning and surgical treatment analysis, and (iv) direct 3D printing of tissues and organs with complete life functions [20].

**Figure 2:**
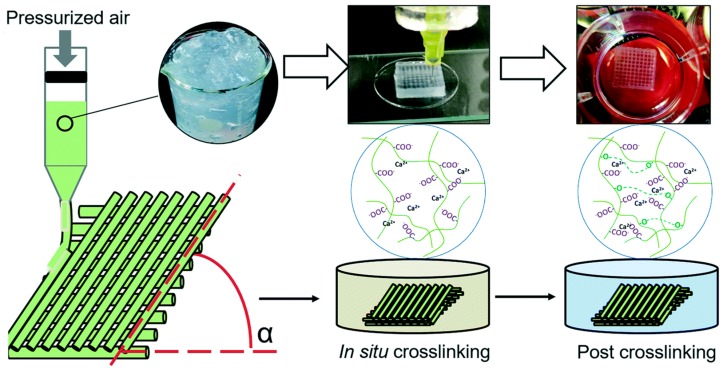
Schematic of the 3D printing process and the two-step crosslinking strategy of nanocellulose hydrogels. Adapted with permission from ref. [26]. Copyright 2018, The Royal Society of Chemistry.

Lately, the term ‘3D bioprinting’ has been increasingly used, which refers to the 3D printing of structures using biocompatible inks (commonly called bio-inks); consisting of biomaterials to be fabricated, living cells, and essential nutrients [21,22]. 3D bioprinting of hydrogels generally follows three steps: (i) design or creation of the model, (ii) printing using bio-inks and (iii) in-situ and/or a post-printing cross-linking process to stabilize the printed structures [23]. A schematic of 3D printing for nanocellulose-based hydrogels is shown in Figure 2. Printability of the ink through the micro nozzle is an important factor that governs the quality of the fabricated structures, and is characterized by the ink’s rheological properties to flow and maintain its printed shape, preventing single filament deformation [19]. Moreover, biocompatible inks that can crosslink at body temperature in a short time, with a low photoinitiator concentration and/or requiring low intensity UV light are generally considered attractive materials for 3D bioprinting [24,25]. 

## 4. 3D Bioprinted Nanocellulose-Based Hydrogels: Properties and Biomedical Applications 

**Figure 3:**
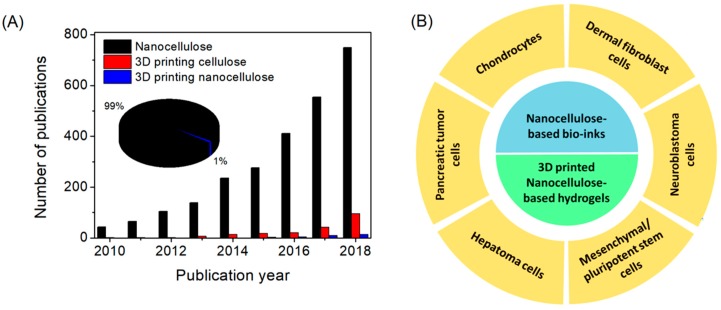
(**A**) Illustration of the annual number of scientific publications using the search terms “Nanocellulose”, “3D printing cellulose”, and “3D printing nanocellulose”. Data analysis was completed using Scopus search system on 28 March 2019. (**B**) Different types of cell lines demonstrated for viability and proliferation on 3D bioprinted nanocellulose-based hydrogels.

Although research activities involving nanocellulose have grown exponentially over the last decade, 3D printing of nanocellulose-based materials is still in its infancy (Figure 3A). This section highlights the recent (last five years) developments and challenges in the 3D bioprinting of nanocellulose-based hydrogels, and the in vitro and in vivo tissue engineering applications of the resultant printed constructs. The ink preparation methods, 3D printing conditions, crosslinking methods, mechanical properties, biodegradability, cellular viability; and attachment and proliferation of the printed objects are discussed wherever possible.

3D printing of geometrically stable, pristine nanocellulose hydrogels that also remain stable after drying still remains a challenge [25]. At low concentrations (1–2%), CNFs are able to entangle with each other to form hydrogel networks that have crucial properties of a 3D printable ink, such as shear thinning (non-Newtonian behavior of fluids whose viscosity decreases under shear strain), strong thickening and sufficiently high yield stress [27]. Moreover, the introduction of charged functional groups to the CNF interface makes the colloidal stability of CNF-based hydrogels very high and it keeps inks viable for a long time [28]. On the other hand, CNC-reinforced inks, designed for 3D printing, may offer advantages over the semi crystalline CNFs because higher solid loadings may be achieved at a given viscosity and storage modulus due to the absence of physical entanglements [29]. To date, there are only a few reports that have successfully demonstrated both 3D printing and mammalian cell viability and proliferation on the resultant printed stable pristine CNF hydrogels. One of the successful methods involved the 3D-printing of TEMPO-oxidized CNF hydrogel scaffolds based on double network cross-linking; first, by in situ CaCl_2_ cross-linking and, second, by post-printing chemical cross-linking with 1,4-butanediol diglycidyl ether. Scaffolds were successfully printed from 1 wt % CNF ink, and the mechanical strength of the 3D-printed hydrogels was tunable in the range of 3 to 8 kPa [26]. Cytocompatibility tests demonstrated that the fabricated scaffolds supported human dermal fibroblast cells proliferation, which improved with increasing scaffold rigidity [26]. However, there is a paucity of reports available on successful 3D printing and mammalian cell viability tests on stable pristine CNC and BNC hydrogels. 

In order to improve the printability (rheological properties) of nanocellulose-based inks and printed shape fidelity, ink formulations using auxiliary materials, such as naturally-derived polymers, including alginates, hyaluronic acid, gelatin, etc., have been applied [25,30]. Moreover, auxiliary materials can also improve the performance of formed composite hydrogels through particle-polymer interfacial interactions (electrostatic, van der Waals force, and hydrogen and covalent bonds) and energy dissipation [31]. A summary of 3D-printed nanocellulose-based hydrogels and their mammalian cell viability (Figure 3B) are provided in Table 1.

**Figure 4:**
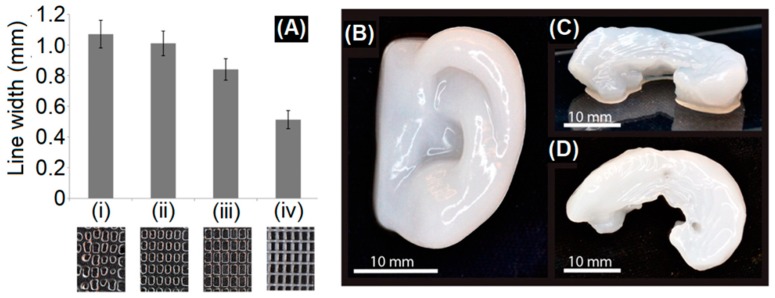
(**A**) Line width measurements of 3D-printed large grids with alginate inks: (i) 2% alginate, (ii) 3% alginate, and (iii) 4% alginate, compared to (iv) Ink9010 (2.25% CNFs + 0.25% alginate). The photos below the graph show the printed grids and their different line resolutions. Small grid printed with (**B**) 3D printed human ear and (**C**,**D**) sheep meniscus with Ink8020 (2% CNFs + 0.5% alginate). (**C**) Side view and (**D**) top view of the sheep meniscus. Adapted with permission from ref. [33]. Copyright 2015, American Chemical Society.

Among natural polymers used in bio-ink formulation; alginate, a low-cost polysaccharide is the most widely used auxiliary material that has demonstrated excellent printability, biocompatibility, and ionic (Ca^2+^) cross-linking functionality [32]. In 2015, Markstedt et al. [33] successfully 3D bioprinted a human ear and sheep meniscus shaped structures using bio-inks containing CNF/alginate blends (90/10, 80/20, 70/30, 60/40) and human chondrocytes (Figure 4). The 3D bioprinted structures were cross-linked by CaCl_2_ and the cross-linking properties were reportedly controlled by varying the proportion of alginate to CNFs without influencing the viscosity; and hence, the printability. The authors also reported an increase in cell viability in the printed constructs after 7 days compared to Day 1 [33]. Martínez et al. [34] 3D bioprinted CaCl_2_ cross-linked auricular and lattice-structured constructs from bio-inks containing a CNF/alginate blend (2/0.5) and human nasal chondrocytes (hNCs) or rabbit auricular chondrocytes (rACs). The cell-laden constructs exhibited an increase in cell viability and proliferation during in vitro culture (28 days), and supported the redifferentiation of hNCs and neo-synthesis of cartilage-specific extracellular matrix components. In vivo chondrogenesis in a 3D bioprinted human cell-laden hydrogel construct cross-linked by CaCl_2_ has been demonstrated by Möller et al. [35]. The cell-laden construct was fabricated from a commercial bio-ink (CELLINK Bioink, Sweden) containing CNFs and alginate. Three groups of printed constructs, encompassing (1) hNCs, (2) human bone marrow–derived mesenchymal stem cells (hBMSCs), and (3) co-culture of hNCs and hBMSCs (20/80) were tested against cell-free scaffolds. The constructs demonstrated compressive stress in the range of 15–39 kPa at 40% strain and maintained their structural integrity after 60 days of implantation. Among the tested group, the co-cultured group showed a more pronounced cell proliferation and enhanced deposition of human collagen II, promising a future application in reconstructive surgery [35]. 

Lately, hydrogels made from alginate sulfate have been shown to promote chondrocyte proliferation while maintaining the expression of chondrogenic markers [36]. Müller et al. [37] compared the 3D bioprinting and bovine chondrocyte cell viability of CNF/alginate sulfate (1.36/0.5) with that of CNF/alginate (1.36/1) bio-ink. CNF/alginate sulfate were 3D printed with high shape fidelity and cross-linked with CaCl_2_. The CNF/alginate sulfate hydrogel discs showed reduced viability values at Day 1, which was suspected due to a yet unknown interaction between CNF and alginate sulfate to exhibit negative side effects. However, the cell viability of hydrogels improved to the same levels as the other conditions at Day 28 and showed mitogenic and collagen II synthesis [37]. 

Hyaluronic acid (HA) or hyaluronan, one of the chief components of the extra-cellular matrix (ECM), is an anionic, nonsulfated glycosaminoglycan widely used in the design of engineered hydrogels due to its biofunctionality [38]. Recently, Henriksson et al. [39] compared the 3D bioprinting and mouse mesenchymal stem cell viability of CNF/HA (80/20) with that of CNF/alginate (80/20) bio-ink. The CNF/HA construct was crosslinked by H_2_O_2_, whereas the CNF/alginate construct was crosslinked by CaCl_2_. The 3D bioprinted scaffolds showed excellent cell viability (95%) with compression stress in the range of 19–55 kPa at 40% strain. Moreover, the gene expression of the adipogenic marker genes increased 2.0–2.2 fold for cells in the 3D bioprinted constructs when compared with 2D cultured cells [39]. The potential of the systems have also been demonstrated for human pluripotent stem cells (iPSCs) [40]. 

Conductive CNF/carbon nanotube (80/20) ink used for 3D printing of neural tissue engineering scaffolds was first reported by Kuzmenko et al. [27]. The 3D printed conductive guidelines exhibited an electrical conductivity of 3.8 × 10^−1^ S/cm, upon which the neural cells preferred to attach, proliferate and differentiate. 

Polyurethane (PU), a high performance elastomer, has also been successfully used to 3D print CNF/PU (9/29) biocomposite hydrogel [41]. Transmission electron microscopy images of the construct revealed a ‘skewer-like’ structure, where CNFs were linked to multiple PU nanoparticles. The 3D-printed hydrogels exhibited a compression storage modulus of ~1.57 MPa and demonstrated mouse and human fibroblast cell proliferation [41].

**Figure 5:**
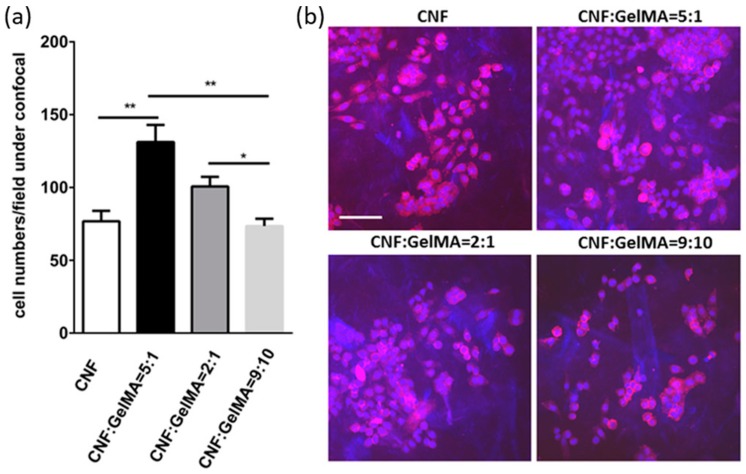
Mouse fibroblast cells were incubated with the indicated 3D matrix in a density of 1 × 10^5^ cells per well. (**a**) The cell proliferation and (**b**) representative confocal images were measured after 3 days of incubation. Scale bar, 50 μm. Bar = mean ± SD; n = 4. * = *p* < 0.1; ** = *p* < 0.01. Matrix hydrogels presenting inks of CNF and CNF/GelMA with weight compositional ratios of 5:1, 2:1, and 9:10. Adapted with permission from ref. [42]. Copyright 2019, American Chemical Society.

Recently, Xu et al. [42,43] demonstrated 3D printing and UV cross-linking of CNF-based inks containing methacrylate derivatives. The CNF/gelatin methacrylate (GelMA) and the CNF/galactoglucomannan methacrylate (GGM) systems were crosslinked with the help of a photoinitiator, Irgacure 2959. The 3D-printed hydrogels demonstrated a compressive Young’s moduli in the range of 2.5–22.5 kPa. The CNF/GelMA system showed mouse fibroblast cell proliferation and viability >90% (Figure 5) [42] and the CNF/GGM system showed human dermal fibroblast and pancreatic tumor cell viability >80% and >60%, respectively [43]. These systems can potentially be applied for wound healing.

Compared to CNF-based systems, the 3D printing of CNC-based composite hydrogels has been less explored. This may be due to the poor shear thinning and gelling properties of CNCs relative to CNFs. Xu et al. [44] exploited the temperature responsiveness of gelatin to 3D print CNC/gelatin composite inks. The fabricated hydrogels demonstrated compressive yield deformation at 20% strain and mouse fibroblast cell viability showed potential for soft tissue regeneration. Wu et al. [45] successfully 3D bioprinted a liver-mimetic honeycomb 3D structure using bio-inks containing CNC/alginate (1/1, 1/2, 2/1, 3/2) and either mouse fibroblast or human hepatoma cells. The bio-ink showed excellent shear-thinning property, extrudability and shape fidelity after deposition. The deposited structures were crosslinked with CaCl_2_ and incubated and cultured for 3 days. The fabricated hydrogels demonstrated shear storage moduli in the range of 8–300 Pa, and cellular viability >67%. 

Recently, Jessop et al. [46] demonstrated 3D bioprinting using a unique blend consisting of CNFs, CNCs, and alginate. This unique blend, bioprinted with human nasoseptal chondrocytes, exhibited nano- and micro-roughness for cellular survival and differentiation, as well as maintaining the most stable construct volume in culture. The 3D bioprinted construct that crosslinked with CaCl_2_ exhibited a compressive Young’s modulus of ~52.6 kPa, and cell viability >71%. Moreover, the chondrocytes demonstrated high metabolic activity post-printing and adopted a rounded chondrogenic phenotype after prolonged culture [46]. However, there is no report available on 3D printing using BNC-based inks.

## 5. Conclusions and Future Perspectives

Nanocellulose, owing to their high surface area, superior strength, tunable surface chemistry, biodegradability, biocompatibility and promotion of cellular interactions and tissue development has emerged as a new generation of nanomaterial for biomedical application. The application of nanocellulose in the 3D bioprinting of cell-laden hydrogels is relatively new and needs further development in terms of specific technical, material and cellular aspects of the process. The process of 3D bioprinting nanocellulose-based hydrogels for tissue engineering and regeneration applications currently involves either the printing of bio-ink and in-situ cross-linking/post-stabilization of printed structure or the printing of stable geometries and post-cell seeding. However, the in vivo degradability of nanocellulose-based scaffolds still remains a challenge as nanocellulose itself does not completely degrade in the human body due to the lack of relevant enzymes [47]. This needs to be addressed as the degradation of a fabricated scaffold after implantation is important to enhance the interaction between host tissues and those encapsulated in the scaffold. A variety of nanocellulose-based bio-inks containing biopolymers, such as alginate, hyaluronic acid, gelatin and mammalian cells, such as chondrocytes, mesenchymal stem cells, fibroblast, etc., have been successfully demonstrated for the 3D bioprinting of nanocellulose-based functional hydrogels. The addition of biopolymers improves the printability, performance and biodegradation of fabricated constructs; and also broadens their biomedical application, including drug delivery and self-healing. It should be noted that the majority of reports are based on bio-inks prepared using CNFs, alginate and chondrocyte cells, owing to the advantage of the shear thinning and gelling properties of CNF/alginate at low concentrations. However, the considerable increase in viscosity of bio-ink with the increase in CNF concentration is a limiting factor. In comparison, viscoelastic bio-inks prepared with high CNC concentrations enabled 3D printing of textured composites with enhanced stiffness along the printing direction [29]. However, the effects of osmolarity caused by high concentration and shear-induced alignment of CNCs on cell viability during 3D bioprinting need to be studied in detail with caution. Moreover, characteristic ribbon-like fibril formation with higher crystalline structure and hydrophilicity that affect the solution viscosity of 3D printing BNC need to be addressed. Nevertheless, further advances in preclinical animal trials are still required to broaden the utilization of nanocellulose-based 3D bioprinted commercial products for tissue engineering and wound healing applications.

## Figures and Tables

**Table 1:** Summary of 3D-printed nanocellulose-based hydrogels; their printing and crosslinking conditions, properties, and biomedical applications.

Hydrogel Composition	Bioink	3D Printing Feed Rate; Nozzle Size; and Pressure	Crosslinking Condition	Mechanical/Electrical Properties	Mammalian Cell Biocompatibility	Biomedical Application	Ref.
CNF	No	8 mm/s; 0.20 mm; 50 kPa	0.01% 1,4-butanediol diglycidyl ether; 50 °C; 2 h	Compressive Young’s moduli: 3.45–7.44 kPa	Human fibroblast cells	Wound healing	[26]
CNF/alginate (90/10, 80/20, 70/30, 60/40)	Yes	5–20 mm/s; 0.30 mm; 20–60 kPa	90 mM CaCl_2_; 10 min	Compressive stress: 22–33 kPa at 30% strain	Human chondrocyte cells; viability—73% (Day 1), 86% (Day 7)	Cartilage tissue engineering	[33]
CNF/alginate (2/0.5)	Yes	5 mm/s; 0.15 mm; 4 kPa	100 mM CaCl_2_; 10 min	-	Human and rabbit chondrocyte cells; viability—96% (human), 99% (rabbit)	Cartilage tissue engineering	[34]
CNF/alginate (CELLINK Bioink, Sweden)	Yes	-	100 mM CaCl_2_; 5 min	Compressive stress: 15–39 kPa at 40% strain	Human chondrocyte and mesenchymal stem cells	Tissue engineering	[35]
CNF/alginate (1.36/1); CNF/alginate sulfate (1.36/0.5)	Yes	0.16–0.41 mm; 6–74 kPa	100 mM CaCl_2_; 12 min	Shear storage modulus: 14.6 kPa	Bovine chondrocyte cells; viability > 85%	Cartilage tissue engineering	[37]
CNF/alginate (80/20); CNF/hyaluronan (80/20, 70/30)	Yes	17–20 kPa	CNF/alginate—100 mM CaCl_2_; 10 min; CNF/hyaluronan—0.001% H_2_O_2_; 5 min	Compression stress: 19–55 kPa at 40% strain	Mouse mesenchymal stem cells; viability—95% (Day 7)	Tissue engineering	[39]
CNF/alginate (60/40); CNF/hyaluronan (80/20)	Yes	10–20 mm/s; 0.30 mm; 20–30 kPa	CNF/alginate—100 mM CaCl_2_; 5 min; CNF/hyaluronan—0.001% H_2_O_2_; 5 min	-	Human pluripotent stem cells	Cartilage tissue engineering	[40]
CNF/carbon nanotube (80/20)	No	10 mm/s; 0.30 mm; 65 kPa	-	Conductivity: 3.8 × 10^−1^ S/cm	Human neuroblastoma cells; viability > 95%	Neural tissue engineering	[27]
CNF/polyurethane (9/29)	No	7–10 mm/s; 0.16 mm and 0.21 mm; 50–200 kPa	-	Compression storage modulus: 1.57 MPa	Mouse and human fibroblast cells	Tissue engineering	[41]
CNF/gelatin methacrylate (5/1, 2/1, 9/10)	No	16–33 mm/s; 0.16 mm and 0.21 mm; 65–80 kPa	0.5% Irgacure 2959; 10 mW/cm^2^ UV (320–390 nm); 5 min	Compressive Young’s moduli: 2.5–5 kPa	Mouse fibroblast cells; viability > 90%	Wound healing	[42]
CNF/galactoglucomannan methacrylate (1/1, 1/2, 1/3)	No	5 mm/s; 0.21 mm	0.5% Irgacure 2959; 10 mW/cm^2^ UV (320–390 nm); 5 min	Compressive Young’s moduli: 2.5–22.5 kPa	Human dermal fibroblast and pancreatic tumor cells; viability > 80% (fibroblast), > 60% (pancreatic)	Tissue engineering	[43]
CNC/gelatin	No	5–15 mm/s; 0.21 mm and 0.41 mm	4 °C and 20 °C; 0.25–24 h	Compressive yield deformation at 20% strain	Mouse fibroblast cells	Tissue engineering	[44]
CNC/alginate (1/1, 1/2, 2/1, 3/2)	Yes	25 mm/s; 0.11 mm; 34–172 kPa	1% CaCl_2_; 10 min	Shear storage moduli: 8–300 Pa	Mouse fibroblast and human hepatoma cells; viability—71% (fibroblast), 67% (hepatoma)	Tissue engineering	[45]
CNC/alginate (4/1); CNF/alginate (4/1); CNC-CNF/alginate (4/1);	Yes	0.61 mm	0.5–1 M CaCl_2_; 2–4 min	Compressive Young’s modulus: 52.6 kPa	Human chondrocyte cells; viability > 71%	Cartilage tissue engineering	[46]

## Abbreviations

3D	three-dimensional
CNC	cellulose nanocrystal
CNF	cellulose nanofiber
BNC	bacterial nanocellulose
TEMPO	(2,2,6,6-Tetramethylpiperidin-1-yl)oxyl
ECM	extra-cellular matrix
hNCs	human nasal chondrocytes
rACs	rabbit auricular chondrocytes
hBMSCs	human bone marrow–derived mesenchymal stem cells
HA	hyaluronic acid
iPSCs	human pluripotent stem cells
PU	polyurethane
GM	gelatin methacrylate
GGM	galactoglucomannan methacrylate
